# Supplementary material for: Incidence and Mortality of Renal Cell Carcinoma after Kidney Transplantation: A Meta-Analysis
Source: J Clin Med. 2019 Apr 17;8(4):530. doi: 10.3390/jcm8040530 (PMC6517974; doi:10.3390/jcm8040530)

Search terms for systematic review.

Databases: Ovid MEDLINE

1. exp transplantation/
2. transplant$.mp
3. 7 or 8
4. kidney.mp
5. renal.mp
6. 10 or 11
7. kidney$.mp
8. renal$.mp
9. bladder$.mp
10. urothelial$.mp
11. urinary$.mp
12. 11 and 12 and 13 and 14 and 15
13. cancer$.mp
14. malignancy.mp
15. malignancies.mp
16. 17 or 18 or 19
17. 16 and 20
18. 10 and 21
19. limit 22 to humans

EMBASE:

('renal cell carcinoma' OR 'kidney cancer') AND 'kidney transplantation' OR

('bladder cancer' OR 'transitional cell carcinoma') AND 'kidney transplantation' OR

('urothelial cancer' OR 'urothelial cancer associated 1') AND 'kidney transplantation'

Cochrane Database

Bladder cancer and kidney transplantation

Bladder cancer and renal transplantation

Transitional cell carcinoma and kidney transplantation

Transitional cell carcinoma and renal transplantation

Urothelial cancer and kidney transplantation

Urothelial cancer and renal transplantation

Kidney cancer and kidney transplantation

Kidney cancer and renal transplantation

Renal cell carcinoma and kidney transplantation

Renal cell carcinoma and renal transplantation

Supplementary Figure 1: Funnel plot evaluating for publication bias evaluating incidence of KTx recipients with RCC

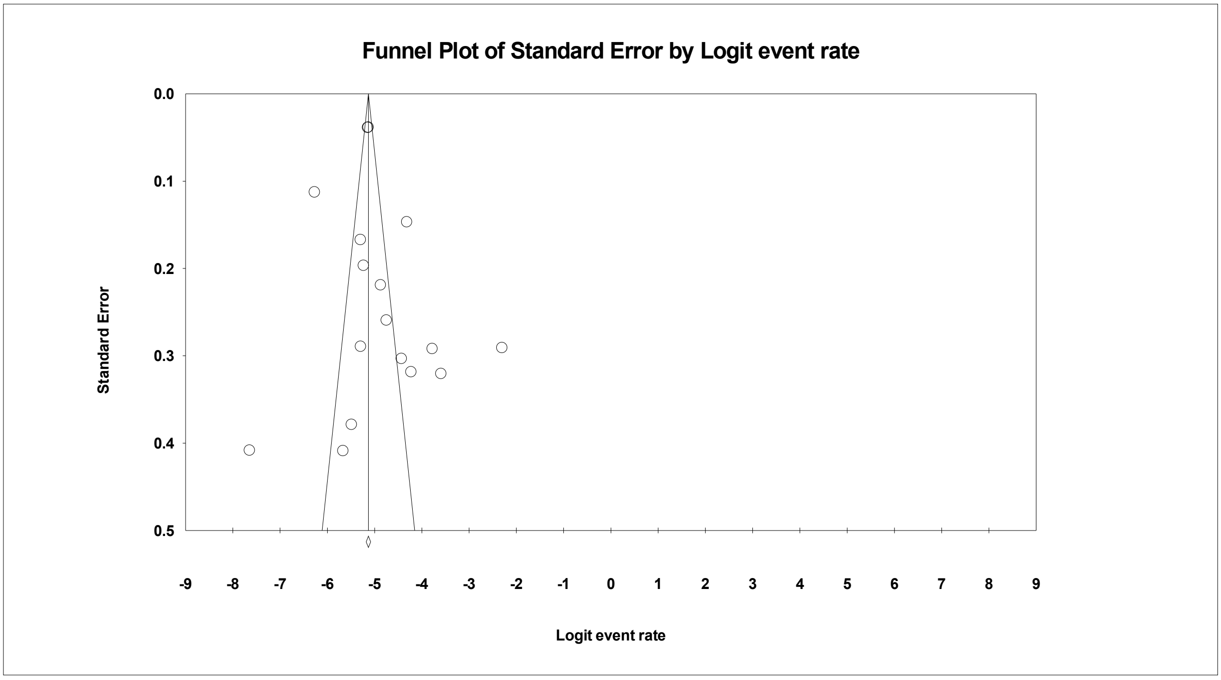

Supplementary Figure 2: Funnel plot evaluating for publication bias evaluating mortality of KTx recipients with RCC

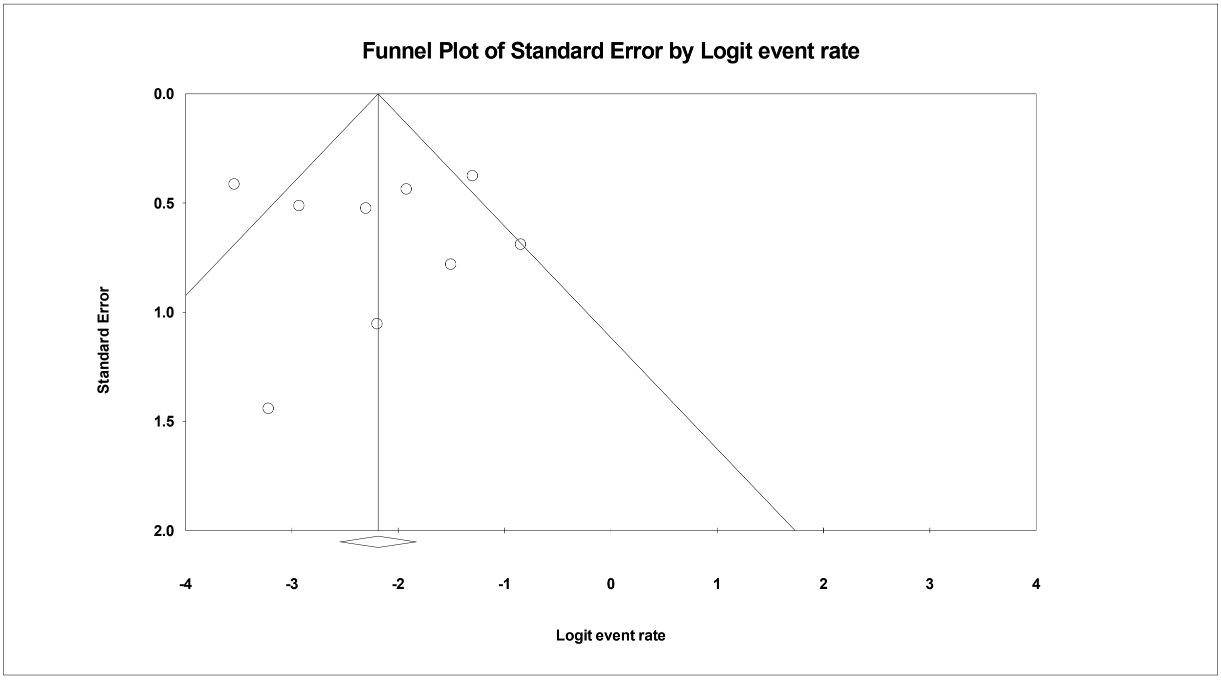

Supplement: Supplementary file 1 [file jcm-08-00530-s001.pdf]
